# Construction of a high-coverage bacterial artificial chromosome library and comprehensive genetic linkage map of yellowtail *Seriola quinqueradiata*

**DOI:** 10.1186/1756-0500-7-200

**Published:** 2014-03-31

**Authors:** Kanako Fuji, Takashi Koyama, Wataru Kai, Satoshi Kubota, Kazunori Yoshida, Akiyuki Ozaki, Jun-ya Aoki, Yumi Kawabata, Kazuo Araki, Tatsuo Tsuzaki, Nobuaki Okamoto, Takashi Sakamoto

**Affiliations:** 1 Faculty of Marine Science, Tokyo University of Marine Science and Technology, 4-5-7, Konan, Minato-ku, Tokyo 108-8477, Japan; 2 Fisheries Research Agency, National Research Institute of Aquaculture, 422-1, Nakatsuhamaura, Minami-ise-cho, Watarai-gun, Mie 516-0193, Japan; 3 Fisheries Research Agency, Goto Branch of Seikai National Fisheries Research Institute, 122-7, Nunoura, Tamanoura-cho, Fukue-shi, Nagasaki 853-0508, Japan

**Keywords:** Yellowtail, BAC library, Linkage map

## Abstract

**Background:**

Japanese amberjack/yellowtail (*Seriola quinqueradiata*) is a commonly cultured marine fish in Japan. For cost effective fish production, a breeding program that increases commercially important traits is one of the major solutions. In selective breeding, information of genetic markers is useful and sufficient to identify individuals carrying advantageous traits but if the aim is to determine the genetic basis of the trait, large insert genomic DNA libraries are essential. In this study, toward prospective understanding of genetic basis of several economically important traits, we constructed a high-coverage bacterial artificial chromosome (BAC) library, obtained sequences from the BAC-end, and constructed comprehensive female and male linkage maps of yellowtail using Simple Sequence Repeat (SSR) markers developed from the BAC-end sequences and a yellowtail genomic library.

**Results:**

The total insert length of the BAC library we constructed here was estimated to be approximately 11 Gb and hence 16-times larger than the yellowtail genome. Sequencing of the BAC-ends showed a low fraction of repetitive sequences comparable to that in *Tetraodon* and fugu. A total of 837 SSR markers developed here were distributed among 24 linkage groups spanning 1,026.70 and 1,057.83 cM with an average interval of 4.96 and 4.32 cM in female and male map respectively without any segregation distortion. Oxford grids suggested conserved synteny between yellowtail and stickleback.

**Conclusions:**

In addition to characteristics of yellowtail genome such as low repetitive sequences and conserved synteny with stickleback, our genomic and genetic resources constructed and revealed here will be powerful tools for the yellowtail breeding program and also for studies regarding the genetic basis of traits.

## Background

Species of yellowtail (family Carangidae) are widely distributed in the world’s ocean and are major target species for fisheries and aquaculture. The Japanese amberjack/yellowtail (*Seriola quinqueradiata*) is one of the most popular fish for consumption in Japan, where about 150,000 tons of farmed fish are produced each year. Although there is a huge market demand, seeds of this fish mostly rely on wild catch and hence artificial seed production is required for stable cultivation and breeding as well as reducing the negative effects of large-scale sampling of seed fish on natural stock. It is well known that using cultured brood fish for seed production reduces the environmental impact and allows the selection of commercially important traits and in such a case, marker-assisted selection (MAS) breeding based on studies regarding quantitative trait locus (QTL) is powerful and cost effective choice. Indeed, QTL studies have been performed in several fishes so far to improve production and life-history traits such as disease resistance and enhance growth rate [1]. To enable the QTL studies, linkage maps are required. In yellowtail, although a female linkage map has been constructed with 180 microsatellite markers [2,3], the number of markers is not sufficient for fine QTL mapping and/or MAS in yellowtail breeding programs. Therefore, a higher-density linkage map is still required.

To isolate simple sequence repeats (SSRs) such as microsatellites and to further investigate the genetic basis of the traits, genomic information is essential. The sequences were isolated from genomic library and of the genomic library, one using bacterial artificial chromosome (BAC) system, called the BAC library, has been frequently used such as to generate whole-genome physical maps by DNA fingerprinting [4], to develop sequence-tagged connectors [5], and to sequence the genome itself [6] because of their insert size capacity, reproducibility and stability as the DNA sample [7]. By integrating BAC clones into linkage maps using BAC-derived sequences such as BAC-end sequences (BESs), BAC library also play important roles in genetic studies and subsequent positional cloning [8]. BAC libraries have been developed in several domestic animals, e.g. cattle [9], pig [10] and sheep [11], and in fishes, salmon [12], catfish [13], rainbow trout, carp, tilapia [14,15], European sea bass [16,17] and barramundi [18] but in yellowtails a BAC library has not yet been constructed.

In this study, to advance yellowtail genomic and genetic resources and for understanding of the genetic basis of several traits, we constructed a high-coverage BAC library, obtained BESs for preliminary survey of the genomic content and constructed comprehensive genetic linkage map of yellowtail.

## Results and discussion

### BAC library construction and BAC end sequencing

The yellowtail genomic DNA content, represented as C-value, was estimated to be 0.7 pg/cell (data not shown) using flow cytometric analysis and hence the genome size was calculated to be approximately 685 Mb. Of 100 randomly selected BAC clones, 71 (71%) contained inserts, indicating that approximately 78,520 (71% of 110,592 clones) clones had an insert. The size distribution of the 71 clones with inserts was from about 20 kb to 220 kb and average insert length was 140.7 kb (data not shown). Therefore, it is estimated that the total length of the yellowtail BAC library insert DNA was approximately 11 Gb and was 16-times larger than the yellowtail genome. It is known that a minimum of 5-10 × coverage across the entire genome is required for a BAC library to be useful for positional cloning, physical mapping, and genome sequencing [19]. Therefore, the yellowtail BAC library is sufficient for further genomic/genetic analysis except for studies regarding W-linked genes because of our ZZ male derived DNA source [20].

By sequencing both ends of randomly-selected 2,960 BAC clones, a total of 5,920 raw reads were obtained, and of those reads, 4,956 reads (2,471 in the SP6 side and 2,485 in the T7 side) were qualified for subsequent repeat identification and BLAST search (GA867436 - GA872391). Total length of the qualified BESs was 3,074,133 bp with an average size of 620 bp, representing approximately 0.45% of the yellowtail genome. The GC content was estimated to be 41.36%, which is almost the same as other fishes (*Takifugu rubripes*: 45.46%; *Gasterosteus aculeatus*: 44.60%; *Oryzias latipes*: 40.46%; *Tetraodon nigroviridis*: 46.43%) (http://esper.lab.nig.ac.jp/genome-composition-database/).

### Preliminary survey of genomic content using BESs

#### Repeat content

The repeat elements were searched and screened from the qualified BESs. Total 211,184 bp (6.87%) of the qualified BESs are assigned to the repeat elements, of which 60,380 bp (1.96%), 40,242 bp (1.31%), 21,563 bp (0.70%) and 76,925 bp (2.50%) were classified as retroelements, DNA transposons, small RNA and simple repeats, respectively (Table 1). Assuming that the BAC library represents the genome of target specie, the abundance of the repetitive sequence in yellowtail genome is lower than the majority of teleost fishes studied so far such as rainbow trout (59.5%) [21], common carp (17.3%) [15], channel catfish (11.9%) [22] and Nile tilapia (14.0%) [23] and comparable to that in *Tetraodon* (6.2%) and in fugu (4.3%) [24].

**Table 1:** Repeat content of the yellowtail BESs

	**Number of elements**	**Length occupied (bp)**	**% of sequence**
Retroelements	284	60380	1.96
SINEs:	57	6098	0.20
Penelope	6	1229	0.04
LINEs:	124	28193	0.92
L2/CR1/Rex	81	16689	0.54
R1/LOA/Jockey	2	112	0.00
R2/R4/NeSL	7	1703	0.06
RTE/Bov-B	24	6124	0.20
L1/01 N4	6	2453	0.08
LTR elements:	103	26089	0.85
BEL/Pao	19	9346	0.30
Ty1/Copia	1	629	0.02
Gypsy/DIRS1	65	14242	0.46
Retroviral	4	881	0.03
DNA transposons	281	40242	1.31
hobo-Activator	73	5609	0.18
Tc1-IS630-Pogo	96	20720	0.67
PiggyBac	13	1887	0.06
Tourist/Harbinger	11	1349	0.04
Unclassified:	15	1165	0.04
Total interspersed repeats:		101787	3.31
Small RNA:	69	21563	0.70
Satellites:	12	1797	0.06
Simple repeats:	1842	76925	2.50
Low complexity:	237	11293	0.37

A total of 1,845 simple sequence repeats (SSRs) were identified from the BESs (Table 1). Of the SSRs, di-nucleotide repeats, particularly AC/GT repeats including CA/TG repeats, were the most abundant (Table 2).

**Table 2:** SSR distribution in the yellowtail BESs

**Repeat**	**Type**	**Number**
Monomer	A/T	234
	G/G	12
Dimer	AC/GT	589
	AG/CT	116
	AT/AT	75
Trimer	AAT/ATT	64
	AGC/GCT	29
	AAC/GTT	20
	ATC/GAT	20
	CTC/GAG	20
	AGG/CCT	16
	Others	41
Tetramer	AAAT/ATTT	48
	AAAC/GTTT	28
	AGAT/ATCT	25
	ACAG/CTGT	16
	Others	145
Pentamer		132
Hexamer		141
Heptamer		63
Octomer		5
Nanomer		5
Decamer		1

#### Homology to other teleost genomes

To identify the homology between yellowtail and other fishes, the yellowtail qualified BESs were subjected to BLASTx and BLASTn searches against eight teleost proteomes and genomes respectively. The highest number of top hits, highest average bit score and % identity were observed in yellowtail-Nile tilapia in both BLAST results (Table 3). Total length of the queries in BLASTx hits between yellowtail and Nile tilapia was estimated to be 198,090 bp indicating that 6.4% of the qualifed BESs was protein coding sequence. The high sequence similarity between yellowtail and Nile tilapia can be explained by their phylogenic positions where they are both assigned in the order Perciformes [25]. In the BLASTn result, the second-highest number of top hit was observed in the yellowtail-stickleback comparison (Table 3). The high sequence similarity between stickleback and species in Perciformes such as striped bass and gilthead seabream has been reported and therefore our data is consistent with the previous observations [26,27].

**Table 3:** Summary of BLAST searches of the yellowtail qualified BESs against eight fish genomes and proteomes

	**BLASTx**				**BLASTn**			
**Species**	**No. of top hits**	E-value*	Bit score*	% identity*	**No. of top hits**	E-value*	Bit score*	% identity*
Atlantic cod	630	2.2E-11	99.1	75.6	720	2.7E-11	125.5	88.8
Medaka	672	1.7E-11	102.7	77.2	1,122	1.6E-11	145.6	88.9
Nile tilapia	768	1.8E-11	1085	80.8	2,162	1.2E-11	172.7	90.0
Platyfish	714	1.9E-11	105.4	77.8	1,339	1.5E-11	147.5	89.2
*Tetraodon*	670	1.6E-11	104.2	77.6	891	1.5E-11	144.9	89.0
Stickleback	704	2.0E-11	107.1	80.4	1,718	1.8E-11	160.4	89.8
Fugu	695	2.1E-11	106.8	78.6	1,136	3.4E-11	131.3	89.6
Zebrafish	640	2.8E-11	98.8	72.5	361	4.2E-11	114.4	87.7

*Average value of the tophits.

### Genetic linkage map

Out of the 743 primer pairs designed from the qualified BESs, 373 primer pairs (27 mononucleotide repeats, 285 dinucleotide repeats, 31 trinucleotide repeats, 26 tetranucleotide repeats, 3 pentanucleotide repeats and 1 hexanucleotide repeat) produced amplicons. In addition to the 464 microsatellite markers derived from the genomic library Ohara et al. developed [2,3], 837 markers in total were included in the yellowtail genetic linkage maps (Additional file 1). No segregation distortion was observed in any markers and hence lethal allele-linked markers were not included in our marker set.

**Figure 1:**
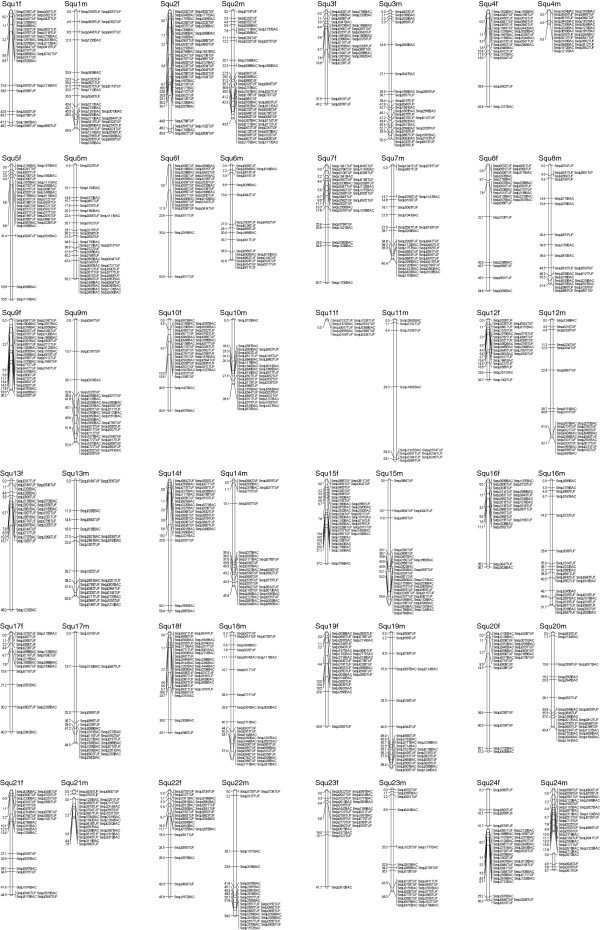
**Yellowtail female (left) and male (right) maps for linkage groups Squ1- Squ24.** Total lengths of linkage groups are expressed in Kosambi cM. BES-derived SSR markers are coded “BAC” after a number, and microsatellite markers developed from genomic library are coded “TUF”.

Resultant yellowtail female and male genetic map consists of 715 and 702 markers including 232 and 271 framework markers, spanning 1,026.65 and 1,057.83 cM Kosambi with an average interval 4.96 and 4.32 cM on 24 linkage groups respectively (Table 4, Figure 1). The number of chromosomes in yellowtail has been reported to be 2n = 48 and hence the SSR markers we developed are distributed throughout the yellowtail genome [28]. The “gaps” observed in Squ21 and 24 in male and both map respectively might be caused by “recombination hot-spots” where recombination occurs frequently (Figure 1). The genome length was estimated to be 1,274.64 (*L*_*1*_) and 1,284.34 (*L*_*2*_) cM in the female and 1,282.35 (*L*_*1*_) and 1,285.45 (*L*_*2*_) cM in the male map by the two different methods respectively (see Materials and Methods). Using formula *c* = 1 – *e*^-2*dn*/*L*^ and estimated genome length *L*, coverage of the female and male map is estimated to be 83.3 to 83.9% respectively (Table 4). Considering the average interval less than 10 cM and the genome coverage, we concluded that the yellowtail genetic map was sufficient for further QTL studies [29].

**Table 4:** Summary of the yellowtail genetic map

	**Female map**					**Male map**				
	**No. of markers**			**Genome length**		**No. of markers**			**Genome length**	
	**All**	**Framework**	**Length (cM)**	***L***_***1 ***_**(cM)**	***L***_***2 ***_**(cM)**	**All**	**Framework**	**Length (cM)**	***L***_***1 ***_**(cM)**	***L***_***2 ***_**(cM)**
Squ1	33	11	48.19	58.11	57.83	34	15	49.28	57.91	56.32
Squ2	49	14	49.27	59.19	56.85	48	18	43.45	52.09	48.56
Squ3	29	10	40.17	50.09	49.10	29	18	54.97	63.60	61.43
Squ4	28	9	42.82	52.74	53.52	25	3	5.57	14.20	11.14
Squ5	38	9	59.51	69.43	74.39	39	17	50.24	58.87	56.52
Squ6	30	5	50.31	60.23	75.47	23	11	42.62	51.26	51.15
Squ7	29	14	50.66	60.58	58.45	32	11	40.41	49.04	48.49
Squ8	26	8	54.83	64.75	70.49	23	12	51.60	60.24	60.99
Squ9	37	10	30.22	40.14	36.94	36	10	52.89	61.53	64.65
Squ10	35	7	40.60	50.52	54.13	36	7	28.60	37.24	38.14
Squ11	10	1	0.00	9.92	0.00	11	6	59.19	67.82	82.86
Squ12	26	12	26.74	36.66	31.60	27	10	52.09	60.73	63.67
Squ13	26	10	48.03	57.95	58.71	25	10	42.63	51.27	52.10
Squ14	34	9	56.36	66.28	70.45	33	11	45.39	54.02	54.47
Squ15	35	14	37.23	47.15	42.96	36	11	55.44	64.07	66.52
Squ16	25	8	36.12	46.04	46.44	27	14	51.66	60.29	59.61
Squ17	20	10	40.32	50.24	49.28	22	7	44.55	53.18	59.39
Squ18	37	10	45.12	55.04	55.15	33	13	57.00	65.64	66.51
Squ19	30	9	40.52	50.44	50.65	26	14	58.45	67.08	67.44
Squ20	22	9	51.21	61.13	64.01	24	10	40.38	49.02	49.36
Squ21	29	12	44.90	54.82	53.06	3	2	1.11	9.75	3.33
Squ21′	N/A	N/A	N/A	N/A	N/A	25	5	7.78	16.42	11.68
Squ22	26	12	45.87	55.79	54.21	26	11	54.03	62.67	64.84
Squ23	25	7	41.67	51.59	55.56	26	10	44.03	52.67	53.82
Squ24	4	4	15.67	25.59	26.12	29	11	17.79	26.42	21.34
Squ24′	32	8	30.31	40.23	38.97	4	4	6.68	15.31	11.13
Average	29	9	41.07	50.99	51.37	27	10	40.69	49.32	49.44
Total	715	232	1,026.65	1,274.64	1,284.34	702	271	1,057.83	1,282.35	1,285.45
Genome coverage *c* (%)				83.56	83.33				83.88	83.81

### Identification orthologous chromosomes with other fishes

**Figure 2:**
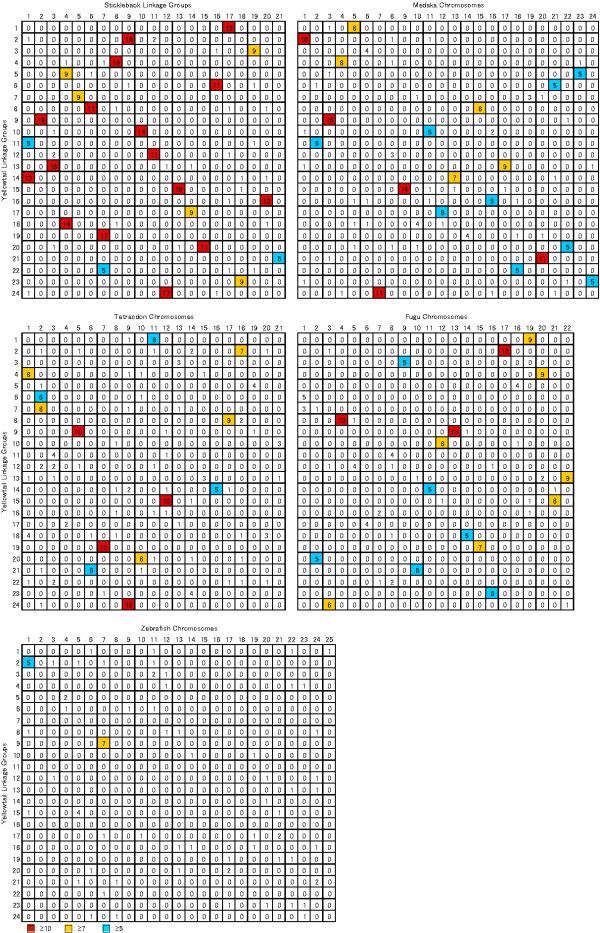
**Oxford grids between yellowtail and five model fish genomes.** Numbers in boxes indicate the number of orthologous gene pairs. Boxes containing more than ten, seven and five orthologous gene pairs are highlighted in red, yellow and blue respectively.

In addition to the BAC or whole genome sequence, comparative genome analysis especially conserved synteny would be helpful for fine-scale QTL analyses and/or understanding the genetic basis of the traits [30,31]. BLAST searches of the 818 mapped yellowtail loci against medaka, *Tetraodon*, stickleback, fugu and zebrafish proved that 25.7, 23.0, 42.2, 24.4 and 9.4% of the loci were mapped to each genome sequence. Oxford grids showed that eighteen linkage group pairs between yellowtail and stickleback retained a one-to-one relationship, and another three stickleback and six yellowtail linkage groups had a one-to-two relationship, implying that chromosomal fusions or breakages occurred after divergence from ancestor of both species (Figure 2). Nevertheless, the result suggests conserved synteny between yellowtail and stickleback and hence the stickleback genome data would be useful as a reference of yellowtail genome.

## Conclusions

We herein constructed a high-coverage BAC library and comprehensive genetic linkage map including BES-derived SSR markers of yellowtail (*Seriola quinqueradiata*). A survey of BESs showed a low frequency of repetitive sequences as much as that of *Tetraodon* and fugu. BLAST searches and Oxford grids against five fish genomes clearly showed conservation between yellowtail and stickleback genome. Generally, a high repetitive sequence frequency hampers chromosome walking and makes the positional cloning difficult [32]. A low frequency of repetitive sequences and relatively small genome size suggest that yellowtail would be an ideal species to study the genetic basis of economically important traits. In addition, conserved genome architecture with stickleback would be helpful for synteny-based identification of new genetic markers and genes in the target genomic segments. We have already started studies regarding several traits such as sex determination and disease resistance [20,33]. We anticipate that the genomic and genetic resources we constructed will be powerful tools for further studies of these traits.

## Methods

### Ethics statements

Field permits are not required for this species in Japan. Since all fish treatments were performed in Goto Branch of Seikai National Fisheries Research Institute of Fisheries Research Agency, fish handling, husbandry and sampling methods were approved by Institutional Animal Care and Use Committee of National Research Institute of Aquaculture (IACUC-NRIA No. 03).

### BAC construction and BAC-end sequencing

The BAC library was constructed according to Katagiri et al. with some modifications [14]. Briefly, at first, approximately 5 × 10^7^ frozen sperm cells taken from one male yellowtail were embedded in agarose plugs, digested with proteinase K overnight at 37°C and stored in 0.5 M EDTA following proteinase K inhibitor treatment until use. The plugs were dialyzed in 0.5 × TE, partially digested with *Mbo*I and size fractionated by pulse-field electrophoresis. The fraction containing 150 to 250 kb genomic DNA was excised from the gel and was recovered as high molecular weight (HMW) genomic DNA. The HMW genomic DNA was then integrated into *Bam*HI site of pBACe3.6 vector and reactions were transfected to *E. coli* DH10B strain. Finally, a total of 110,592 recombinant BAC clones were picked and stored in 288 384-well microtiter plates. The length of the insert DNAs was estimated by analyzing 100 BAC inserts digested with *Not*I.

The BESs were obtained from eight 384-well plates containing 3,072 clones. The BAC DNAs extracted by conventional alkaline lysis method were sequenced from SP6 and T7 sides with BigDye Terminator v3.1 Cycle Sequencing Kit (Life Technologies) following the manufacturer’s instructions and reactions were electrophoresed with Applied Biosystems 3730 DNA Analyzer (Life Technologies). All raw reads were processed using PHRED software with default parameters except for the trimming error probability was set at 0.01 [34,35], and vector and bacterial sequences were masked by CROSS_MATCH implemented in PHRAP software. The masked BESs of more than 100 bp in length, hereafter called “qualified BES”, were extracted using our in-house perl script. The GC content of the extracted BESs was estimated using the geecee program included in the EMBOSS package [36].

### Sequence data analysis

Repetitive DNA elements in the qualified BESs, such as transposable elements and SSRs, were identified and masked using Crossmatch search engine (v1.090518), “teleostei” repeat database implemented in Repbase RepeatMasker Edition (20120418) and RepeatMasker program (see http://www.repeatmasker.org/ for details).

The repeat-masked qualified BESs were subjected to homology search. The eight fish proteome data sets (Atlantic cod: gadMor1.70, *Tetraodon*: TETRAODON8.70, medaka: MEDAKA1.70, Nile tilapia: Orenil1.0.70, platyfish: Xipmac4.4.2.70, stickleback: BROADS1.70, fugu: FUGU4.70 and zebrafish: Zv9.70) were obtained from Ensembl (ftp://ftp.ensembl.org/). Only the longest protein for each gene was extracted and used for in-house database construction. For construction of genomic sequence database, the genomic sequences of stickleback (gasAcu1) and fugu (fr3) were downloaded from UCSC genome browser (http://hgdownload.soe.ucsc.edu/) and others from Ensembl.

BLAST searches were performed with qualified BESs as query with cut-off *e*-value e^−9^. The top hit query-subject pairs were extracted using in-house perl script under the criteria in which if multiple query-subject pairs were observed and were overlapped each other, only the most significant pair was considered significant.

### Development of SSR markers

The SSR motifs and primer pairs in the qualified BESs were searched by WebSat online application (http://wsmartins.net/websat/) with default settings except for product size, which was set to 100–200 bp. For each BAC clone, the SP6-side BES was at first analyzed and if no SSR motif or primer-binding site was found in the sequence, T7-side BES of the same clone was alternatively used. In both sides, the SSR motifs containing over six repeats were considered as real SSRs. In the case where more than two SSR motifs were found in one read, the longest one was used as a representative. Finally, 743 SSRs (89 mono-, 550 di-, 68 tri-, 31 tetra-, 4 penta- and 1 hexa-nucleotide repeats) were selected for primer design.

In addition to the BES-derived SSRs, we also developed microsatellite markers from the genomic library constructed by Ohara et al. [2,3]. The microsatellites containing CA/GT repeat motifs were isolated according to the protocol of Ohara et al. and primers were designed as described above [2,3].

### Mapping panel

The mapping panel consists of ninety progenies produced by artificial fertilization. Parent fish were caught off Goto Island, Nagasaki Prefecture, Japan and reared in a sea cage until they were matured with approximate body weight 7 kg. Human chorionic gonadotropin (ASKA Pharmaceutical) was intramuscularly administered to the parent fish at 600 IU/kg body weight and eggs and sperm were taken at 45 hours after administration. Fertilized eggs were kept in 500 L seawater at approximately 19°C with 0.5-1 L/min aeration until hatching. The juvenile fish were reared in 500 L seawater at 20-25°C until their body length reached 10 cm. The caudal fin was partially clipped from each progeny as the DNA source and kept in absolute ethanol until use. Genomic DNA of each fish was extracted using DNeasy Blood and Tissue Kit (Qiagen) according to manufacturer’s instructions.

### Data acquisition

Genotyping was performed in an 11 μl reaction volume containing 0.5 pmol/μl of unlabelled primer, 0.05 pmol/μl of fluorescence-end-labeled primer with [5’-TET], 1 × buffer, 2.0 mM MgCl_2_, 0.2 mM dNTP, 1.1 μg of BSA, 0.025 U of *EX Taq* DNA polymerase (Takara) and 25 ng of template DNA. PCR was performed on a GeneAmp® PCR System 9700 (Applied Biosystems), and the program conditions were 95°C for 2 min for initial denaturation, followed by 30 cycles of 30 sec at 95°C, 1 min at the annealing temperature (52-55°C), 1 min at 72°C and 10 min at 72°C for final extension. Amplification products were mixed with an equal volume of loading buffer (98% formamide, 10 mM EDTA, 0.05 w/v% bromophenol blue), heated for 10 min at 95°C and then immediately cooled on ice. 2 μl of each sample was loaded onto a 6% PAGE-PLUS gel (Amresco) containing 8 M urea and 0.5 × TBE buffer. Electrophoresis was performed in 0.5 × TBE buffer, and after electrophoresis, the gel was scanned and imaged using a FLA-9000 image scanner (GE Healthcare).

### Linkage map construction

Genotype data obtained above were subjected for linkage analysis for the male and the female meiosis independently. Marker genotypes were analyzed with LINKMFEX ver. 2.3 (http://www.uoguelph.ca/~rdanzman/software.htm). Linkage analysis was performed using genotype data converted to a backcross format. As the grandparent genotypes were unknown, pairwise analyses were performed, and markers were sorted in linkage groups at a minimum LOD score of 4.0. A goodness-of-fit for Mendelian segregation distortion was tested for all alleles using the chi-square test (*p* < 0.05, d.f. = 1). Finally, the marker order was determined and double recombination events were checked with MapManagerQTX version 2.0 [37]. The resultant genetic map was visualized using MapChart version 2.2 [38].

The genome length *L* was estimated using two different methods following Fishman et al. [39]. In the first method (*L*_*1*_), average marker interval was estimated by dividing the summed length of all linkage groups by the number of intervals, and twice the average marker interval was added to each linkage group. In the second method (*L*_*2*_), the length of the each linkage group was multiplied by the factor (m + 1)/(m - 1), where m is the number of framework markers on the linkage groups. Finally, genome coverage *c* of the linkage map was estimated by calculating *c* = 1 - *e*^-2*dn*/*L*^, where *d* is the average interval of markers, *n* is the number of markers, and *L* is the genome length estimated above.

### Identification orthologous chromosomes with other fishes

The flanking sequences obtained from all SSR markers assigned to the yellowtail linkage groups were used for the BLASTn search against genomic sequences of medaka, *Tetraodon*, stickleback, fugu and zebrafish with a cut-off *e*-value of 0.01. The top hit query-subject was extracted using in-house perl script. In the case where multiple hits were obtained, we defined orthology as follows; let us consider only the first, second and third top hit, if query position of the first and second/third top hit is overlapped each other and quotient of *e*-value of the first hit divided by that of the second/third hit is greater than 10^−3^, the hit is considered to be an unclear orthologous pair and rejected. The substantial hits were processed for constructing Oxford grid using Grid Map ver. 3.0a (http://cbr.jic.ac.uk/dicks/software/Grid_Map/).

## Availability of supporting data

All the supporting data are included as additional files.

## Competing interests

The authors declare they have no competing interests.

## Authors’ contributions

KF performed all molecular experiments, constructed the linkage map and drafted the manuscript. TK analyzed all data and drafted the manuscript. WK contributed to data analysis. SK helped SSR marker genotyping. KY performed fish breeding. AO took part in developing the linkage analysis pipeline. JA, YK, KA, and TT provided laboratory facilities and support this program in all steps. NO and TS conceived and overlooked the project and reviewed the manuscript. All authors read and approved the final manuscript.

## Supplementary Material

Additional file 1 **SSR markers in the yellowtail map.** Marker name, linkage group, polymorphic information (1: polymorphic in both female and male, 2: polymorphic in only female, 3: polymorphic in only male), genotype of dam and sire, kind of repeat motif number, repeat type, primer sequences, annealing temperature, PCR product size, and GenBank accession number are shown. Click here for file
